# Efficient Searches in Protein Sequence Space Through AI-Driven Iterative Learning

**DOI:** 10.3390/ijms26104741

**Published:** 2025-05-15

**Authors:** Ignacio Suárez-Martín, Valeria A. Risso, Rocío Romero-Zaliz, Jose M. Sanchez-Ruiz

**Affiliations:** 1 Unidad de Excelencia de Química Aplicada a Biomedicina y Medioambiente (UEQ), Departamento de Química Física, Facultad de Ciencias, Universidad de Granada, 18071 Granada, Spain; suarezmartin@ugr.es (I.S.-M.); vrisso@ugr.es (V.A.R.); 2 Centro de Investigación en Tecnologías de la Información y las Telecomunicaciones (CITIC-UGR), Universidad de Granada, 18071 Granada, Spain; 3 Departamento de Ciencias de la Computación e Inteligencia Artificial, Escuela Técnica Superior de Ingenierías Informática y de la Telecomunicación, Universidad de Granada, 18071 Granada, Spain; 4 Andalusian Research Institute in Data Science and Computational Intelligence (DaSCI), Universidad de Granada, 18071 Granada, Spain

**Keywords:** enzyme engineering, viral protein evolution, focused library screening

## Abstract

The protein sequence space is vast. This fact, together with the prevalence of epistasis, hampers the engineering of novel enzymes through library screening and is a major obstacle to any attempt to predict natural protein evolution. Recently, specialized methodologies have been used to determine fitness data on ~260,000 sequences for the gene of the enzyme dihydrofolate reductase and antibody affinity data for all combinations of the mutations present in the receptor-binding domain (RBD) of the Omicron strain of SARS-CoV-2 (~30,000 variants). We show that upon iterative training on a total of just a few hundred variants, various state-of-the-art AI tools (multi-layer perceptron, random forest, and XGBoost algorithms) find very high fitness variants of the enzyme and predict the antibody evasion patterns of the RBD. This work provides a basis for efficient, widely applicable, low-throughput experimental approaches to assess viral protein evolution and to engineer enzymes for biotechnological applications.

## 1. Introduction

The protein sequence space is the mathematical space of all possible sequences. The full space for a given sequence length (L) is enormous: 20^L^ amino acid sequences encoded by 4^3L^ gene sequences. Searching the sequence space for useful or relevant protein properties is a daunting task, in particular because the prevalence of epistasis (i.e., strong non-additivity of mutational effects) makes it impossible to describe the values of the relevant property for all sequences in terms of a comparatively small number of individual mutational effects [1,2,3,4,5]. Indeed, laboratory directed evolution, the most generally applicable approach to protein engineering [6], is typically sluggish and may require many rounds of effort-intensive library screening to achieve the desired levels of the targeted properties [7]. This obviously reflects the vastness of the protein sequence space and the fact that most sequences do not encode for proteins with biotechnologically useful properties. As a result, there has been considerable interest in the development of computational and experimental methodologies to focus directed evolution to small regions of the sequence space that are predicted to be particularly relevant for the targeted protein property [8,9,10,11,12,13,14,15,16,17], including methodologies based on machine learning (see Yang et al. [18] and references quoted therein).

Here, we show that efficient searches in regions of the protein sequence space spanning at least a few hundred thousand variants can be performed using simple AI tools that are iteratively trained on a total of only a few hundred variants. The notion that machine learning tools can be iteratively used to increase the efficiency of directed evolution has been very recently proposed [18]. In this work, we take advantage of a recent study in which a specialized methodology has been used to determine fitness data on ~260,000 sequences for the gene of the enzyme dihydrofolate reductase [19] to systematically explore the approach. Furthermore, we provide evidence that the AI iterative approach is also useful for sequence space search problems beyond the directed evolution of enzymes. Specifically (see further below in this Introduction section), we demonstrate its potential applicability to the prediction of antibody evasion patterns.

**Figure 1:**
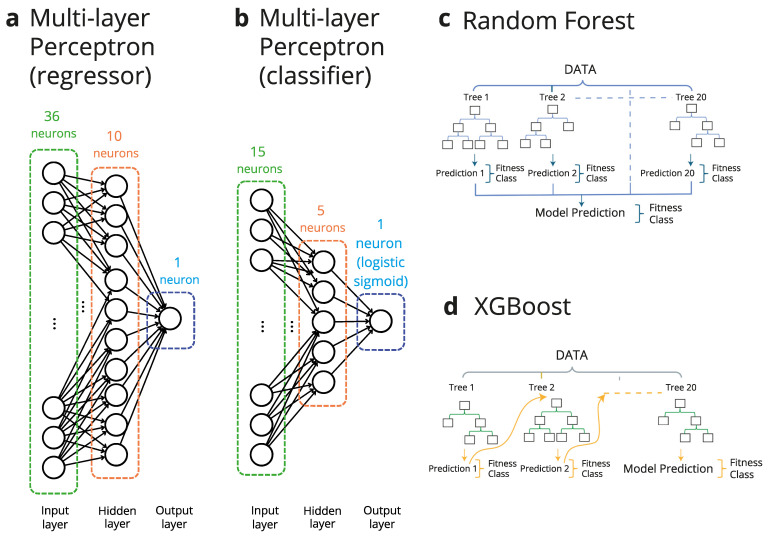
Types of AI tools used in this work. Several AI tools were used in this work either as regressors or as classifiers to search the protein sequence space. (**a**) Multi-layer perceptron used as a regressor to search the sequence space of the enzyme dihydrofolate reductase. Since we use one-hot encoding, there are 36 neurons in the input layer corresponding to the 4 possible nucleotides at the 9 targeted positions in the enzyme gene (see text for details). There is a single, fully connected intermediate hidden layer of 10 neurons. (**b**) Multi-layer perceptron used to search the sequence space of the receptor-binding domain (RBD) of the SARS-CoV-2 virus. It includes as an output a single neuron with a logistic sigmoid activation function, in such a way that it acts as a classifier that allocates variants to two classes: “evasion” and “no evasion”. The input layer has 15 neurons, corresponding to the 15 mutations that separate the RBD of the Omicron BA.1 strain from that of the parent Wuhan Hu-1 strain. There is a single, fully connected intermediate hidden layer of 5 neurons (see text for details). (**c**) Random forest algorithm, which combines 20 decision trees to generate an output. (**d**) XGBoost algorithm, which provides a regularized gradient boost to the random forest. Note that both the random forest and XGBoost algorithms were used as regressors (to search the enzyme sequence space) and classifiers (to search the RBD sequence space).

**Figure 2:**
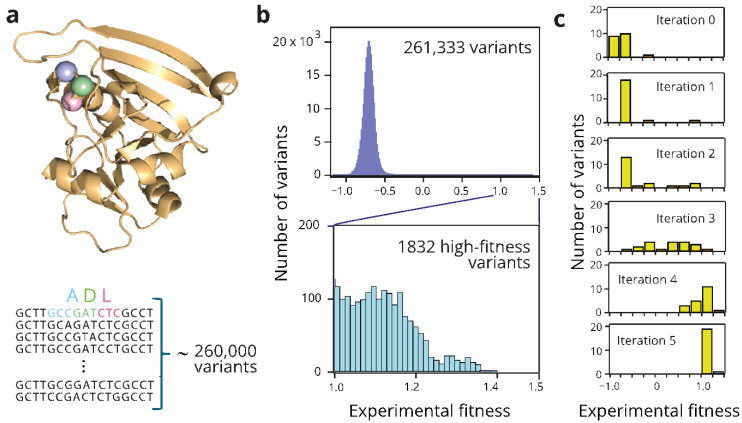
Fitness distribution for variants of dihydrofolate reductase. Experimental fitness values for the enzyme variants in a large combinatorial library were determined [19] from the DNA variant frequencies in the organism’s population and are given in a natural logarithm scale with the wild-type enzyme taken as reference and assigned a fitness value of zero. (**a**) The 3D structure of dihydrofolate reductase (PDB ID 6XG5) and section of the enzyme gene sequence including the 9 positions that were randomized [18] to generate a library of ~260,000 variants. These 9 positions in the gene encode for 3 amino acid residues in the enzyme active site (A, D, and L in the wild-type enzyme). The locations of the three residues are shown in the 3D structure. (**b**) Distribution of fitness values for the ~260,000 variants generated, as shown in (**a**). The logarithmic fitness scale is defined in such a way that the fitness of the wild-type enzyme is zero. Most variants display impaired fitness, while only about 0.7% of the variants have a much-enhanced fitness value above unity (blow-up shown in the lower panel). (**c**) Illustrative example of an AI-driven iterative learning search of the library. A multilayer perceptron (Figure 1a) is initially trained with 20 randomly chosen variants (iteration 0). The sequence/fitness data for the top 20 predictions are then incorporated to the training set for the next round of training, thereby initiating the iterative learning procedure. The plots show the distribution of the experimental fitness values for the top 20 variants of the training set in the successive iterations.

**Figure 3:**
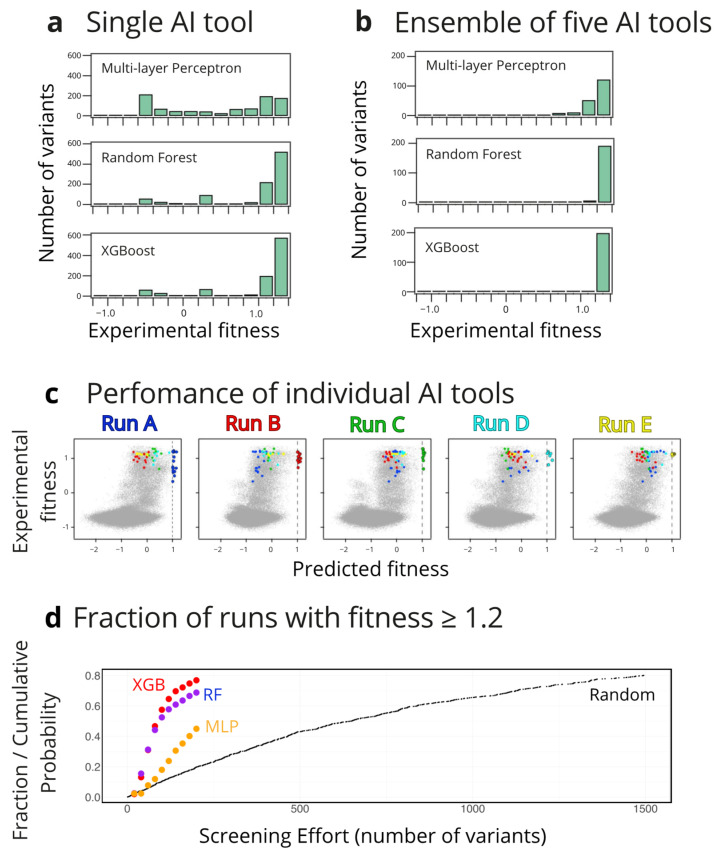
Finding high-fitness variants of dihydrofolate reductase through AI-driven iterative learning. The AI tools displayed in Figure 1 were iteratively trained on small sets of variants randomly selected from the original set of experimental data [19]. In order to test the robustness of the approach, a large number of replica analyses were performed. (**a**) Distribution of best (higher experimental fitness value) variants in the 5th iteration from 1000 search replicas using three different AI tool (Figure 1a,c,d). (**b**) Distribution of best (higher experimental fitness value) variants in the 5th iteration for ensembles of 5 AI tools of the same type in each case. Here, 200 replicas of the ensemble search were performed for each AI tool. (**c**) individual AI tools do not achieve an accurate description of the fitness value for the whole library but rather they locate different high-fitness regions in the sequence space. This is illustrated by the plots of experimental versus predicted fitness for 5 searches based in multi-layer perceptrons in the 5th iterative round. Each run predicts a certain number of high-fitness variants, which differ from the high-fitness variants predicted by the other runs. To make this fact visually clear, the high-fitness predictions from each perceptron are color-coded. (**d**) Comparison of the efficiency of the AI iterative approaches with that afforded by a random library screening approach. For each approach, many replica runs were performed in such a way that the probability of finding at least one variant of very high fitness (defined for the purpose of this calculation as fitness ≥ 1.2; see Figure 3b) can be estimated. These probabilities are plotted versus the screening effort, i.e., the number of variants screened. It is visually apparent that random library screening (labelled “random”) is much less efficient than the AI iterative approaches based on the multilayer perceptron (MPL), random forest (RF), and XGBoost (XGB) algorithms.

The state-of-the-art AI tools we have used (see Figure 1) are as follows: (1) a multi-layer perceptron [20], a feedforward neural network (Figure 1a); (2) a random forest algorithm [21], which combines several decision trees to generate a result (Figure 1c); (3) the XGBoost algorithm [22], which provides a regularized gradient boost to the random forest (Figure 1d). When used as regressors, we find that the three AI tools identify very rare variants of highly enhanced fitness, even after only a few rounds of iterative learning involving a total of just a few hundred sequences, i.e., a number much smaller than the size of the explored region of the sequence space. This result is particularly remarkable, since analyses of the original data [19] supported the prevalence of epistasis, including higher-order epistasis (i.e., non-additivity of the effects of more than two mutations), which brings about a rugged, difficult-to-search fitness landscape.

Beyond the obvious implications for protein engineering, the fact that the protein sequence space is vast is also a major obstacle to any attempts to predict viral protein evolution on the basis of laboratory studies on the relevant biomolecular interactions. This is because viruses have a capability to search the protein sequence space that currently available experimental approaches cannot match. This capability results not only from the high viral mutational rates but also from the huge numbers of virions during an infection. For instance, the number of virions in each person infected by SARS-CoV-2 has been estimated to be 10^9^–10^11^ at peak infection [23]. Overall, viruses may efficiently search the protein sequence space, and when faced with new challenges they may come up with solutions involving large numbers of mutations. For instance, the original Omicron strain of SARS-CoV-2 evades many antibodies against earlier variants of the virus and has 15 mutations in the receptor-binding domain of the spike protein, which is the main target of neutralizing antibodies [24], and variants with many additional mutations are emerging [25,26]. Recently, Moulana et al. [24] used a specialized methodology to determine antibody affinity data for all combinations of the mutations present in the receptor-binding domain (RBD) of the Omicron strain of SARS-CoV-2, i.e., about ~30,000 variants. In order to test the generality of our approach, we used the iterative learning approach using state-of-the-art AI tools depicted in Figure 1 to search the RBD sequence space. Here, our analyses aimed at achieving a reliable classification of the variants in terms of antibody evasion versus non-evasion; that is, the AI tools were used as classifiers (Figure 1b–d). We found that upon iterative training on a total of just a few hundred variants, the AI tools correctly assigned most variants to the evasion or non-evasion classes for three different neutralizing antibodies. Again, this is a remarkable result, since epistasis plays an important role in determining antibody affinity in this case [24], and this should bring about a rugged, difficult-to-search landscape.

## 2. Materials and Methods

### 2.1. Searching the Sequence Space of the Enzyme Dihydrofolate Reductase

*Data Preprocessing:* Codon sequences and their corresponding fitness values were extracted from the Zenodo’s repository: https://zenodo.org/records/8229020 (accessed on 2 November 2024). The dataset fitness_data_wt.csv contains two key columns: **SV**, which includes codon sequences, and **m**, representing fitness values. Each codon sequence was transformed into a one-hot encoded numerical representation to facilitate model training.

A one-hot encoding technique was applied to the gene sequences for them to be used by the AI tools selected. The encoding was performed as follows: the gene sequence at the three variable amino acid positions, i.e., a sequence of 9 consecutive nucleotide positions, was represented as a binary matrix measuring 4 × 9. Each column corresponded to one of the four possible nucleotide bases (A, C, G, T) at a specific position. The binary matrix was then flattened into a 1D array with a length of 36 for compatibility with our computational models.

*Training and Iterative Enrichment:* A random forest regressor, an XGBoost regressor, and a multi-layer perceptron regressor model were employed to predict fitness values. In each case, the model was trained iteratively over 20 iterations, with 20 initial randomly selected training sequences and 20 additional sequences added at each iteration. The iterative process was conducted as follows. (1) Model initialization and training: At each iteration, the regressor model was trained on the current training dataset, consisting of one-hot encoded nucleotide sequences and their corresponding experimental fitness values. (2) Prediction and selection: Fitness predictions were made for all sequences in the validation subset. The top 20 sequences with the highest predicted fitness values were selected and added to the training dataset.

*Replica Runs:* To explore the robustness, searches in the sequence space were repeated 1000 times. Each run utilized a different random seed for splitting the dataset into training and validation subsets. After each run, the trained model was saved using the joblib library for further analyses.

*Implementation Details.* All computations were conducted using Python 3.9. The following libraries were utilized: Pandas [27] for data manipulation, NumPy [28] for numerical operations, scikit-learn [29] for model design and performance evaluation, Joblib for model serialization, and the XGBoost package.

The XGBoost model was implemented using the XGBRegressor function from the XGBoost library using default parameters, except for n_estimators (number of trees), which was set to 20.

The random forest model was implemented using the RandomForestRegressor function from the scikit-learn library using default parameters, except for n_estimators (number of trees), which was set to 20.

The multi-layer perceptron model was implemented using the MLPRegressor function from the scikit-learn library using default parameters except for hidden_layer_sizes, where only one hidden layer was used, and its dimensionality was set to 10. The maximum number of epochs was set at 200, using 0.0001 as the tolerance in the optimization process. We used Adam’s method [30] as a solver for the weight optimization and ReLU as the activation function [31]. The mean squared error was used as the loss function.

For all models, a single run takes a few seconds using a laptop equipped with a 13th Gen Intel^®^ Core^TM^ i7-13700H CPU.

### 2.2. Searching the Sequence Space of the Receptor-Binding Domain of SARS-CoV-2

Data Preprocessing: The dataset used in this study was obtained from the GitHub repository (https://github.com/desai-lab/omicron_ab_landscape, accessed on 2 November 2024) that accompanies the paper by Moulana et al. [24]. In that repository, the following files can be found: lasso_prediction_REGN10987_aic_opt_order_biochem.txt lasso_prediction_CoV555_aic_opt_order_biochem.txt lasso_prediction_CB6_aic_opt_order_biochem.txt.

We extracted the data from the columns genotype and actual Kd in these files. The genotype column represents the protein sequence encoded in a 15-element vector, where a value of 1 in an element means that the Omicron strain amino acid is present in the corresponding position, while having a 0 means that the amino acid in the RBD of the Wuhan Hu-1 strain is present. The corresponding value in the actual Kd column is their experimental value for -log(Kd).

To classify genotypes into two classes, we used the following criteria: (1) Genotypes with -log(Kd) values greater than 6.0 were assigned to class 1 (no evasion). This is equivalent to an antibody dissociation constant smaller than 1 micromolar. (2) Genotypes with -log(Kd) values less than or equal to 6.0 or NaN (not a number) were assigned to class 0 (evasion). This is equivalent to an antibody dissociation constant higher than 1 micromolar.

*Computational Design:* The analysis was conducted across 500 independent runs to explore the statistical robustness of the analysis. Each individual run involved the following steps. (1) Initial training and validation sets: The dataset was randomly split into an initial training set of 20 genotypes and a validation set containing the remaining genotypes. (2) Iterative training process: The iterative learning process was conducted for 20 iterations. During each iteration (A) a classifier model was trained, (B) the classifier’s accuracy was measured on the validation set, and (C) the classifier predicted the class labels for the entire dataset in each iteration.

*Genotype Selection for Training:* To iteratively expand the training set while maintaining diversity we used the follows methods. (1) Hamming distance constraint: (a) A Hamming distance threshold was enforced to ensure that new genotypes added to the training set were sufficiently different from those already included. Initially, the threshold was set to 3. Note that in this context, the Hamming distance [32] is equivalent to the number of mutational changes. (b) If fewer than 20 genotypes met this criterion after 100,000 attempts, the threshold was relaxed to 2, and the search continued. (2) Random selection: If the above steps failed to identify 20 genotypes, the remaining genotypes were randomly selected from the validation set.

*Implementation Details:* The XGBoost model was implemented using the XGBClassifier function from the XGBoost library using default parameters, except for n_estimators (number of trees), which was set to 20.

The random forest model was implemented using the RandomForestClassifier function from the scikit-learn library [29] using default parameters, except for n_estimators (number of trees), which was set to 20.

The multi-layer perceptron model was implemented using the scikit-learn library‘s MLPClassifier function using default parameters, except for hidden_layer_sizes, where only one hidden layer was used, and its dimensionality was set to 5. The maximum number of epochs was set at 200, using 0.0001 as tolerance in the optimization process. We used Adam’s method [30] as the solver for the weight’s optimization and ReLU as the activation function for the hidden layer. A logistic sigmoid function was used in order to set the binary classification.

All pipelines were written in Python. The key libraries included pandas (McKinney, 2010) for data manipulation, numpy [28] for numerical operations, and scikit-learn for data splitting and metric computation. Random seeds were set in each run to ensure reproducibility.

For all models, a single run takes a few seconds using a laptop equipped with a 13th Gen Intel^®^ Core^TM^ i7-13700H CPU.

### 2.3. Additional Details About the Implementation of the AI Models

To facilitate the reproducibility of our computational approach, we present here relevant details of the implementation of the AI models, including the hyperparameter selection and optimization procedures.

*Random Forest:* We used the default scikit-learn implementation process for both the regressor and the classifier. The only input modification was setting n_estimators = 20, i.e., the number of trees to be used, in order to avoid overfitting as much as possible. Gini was the function we used in order to generate the splits. Hyperparameters: n_estimators = 20 (explicitly set). Default parameters: max_depth = none (nodes expand until leaves are pure), min_samples_split = 2, min_samples_leaf = 1, max_features = “auto” (regression: max_features = 1.0; classification: max_features = “sqrt”), bootstrap = true. Criterion: “squared_error” (regression) or “gini” (classification). Optimization: no hyperparameter tuning was applied to maintain simplicity and reproducibility.

*XGBoost:* We used the default XGBoost implementation process for both the regressor and the classifier. The only input modification was setting n_estimators = 20. Hyperparameters: n_estimators = 20 (explicitly set). Default parameters: learning_rate = 0.3 (eta), max_depth = 6, subsample = 1, colsample_bytree = 1, objective: “reg:squarederror” (regression) or “binary:logistic” (classification), booster = “gbtree”, gamma = 0, reg_alpha = 0 (L1 regularization), reg_lambda = 1 (L2 regularization). Optimization: no hyperparameter tuning (e.g., grid/random search) was performed; the study focused on baseline performance with minimal customization.

*Multi-Layer Perceptron:* We used the default scikit-learn implementation process for both the regressor and the classifier. Default parameters: one single hidden layer for the virus study with a size of 5, one single hidden layer for the protein study with a size of 10, activation = “relu”, solver = “adam”, alpha = 0.0001 (L2 regularization term), learning_rate = “constant” (initial rate 0.001), max_iter = 200, batch_size = “auto”, early_stopping = false. Optimization: no custom tuning was performed; default settings were retained to ensure a fair comparison with other baseline models.

### 2.4. On the Possibility of Overfitting

Overfitting was a risk, in particular in the first steps of our iterative process in which very small training sets were used. However, a number of safeguards were implemented in our methodologies. These are briefly listed below.

XGBoost: L1/L2 regularization: reg_alpha = 0 (L1) and reg_lambda = 1 (L2) penalizes extreme weights. Tree complexity control: max_depth = 6 and gamma = 0 limit tree depth and node splitting, respectively.

Random Forest: Feature subsampling: max_features = “auto” (sqrt of features for classification) decorrelates trees. Bootstrap aggregation: training on bootstrapped subsets (bootstrap = True) introduces diversity. Shallow trees: max_depth = none, trees grow until pure but the ensemble averages predictions.

Multi-Layer Perceptron: L2 regularization: alpha = 0.0001 (small but non-zero weight decay). Early stopping: although early_stopping = false by default, the fixed max_iter = 200 limits training epochs. Activation function: ReLU’s sparsity can reduce overfitting compared to saturating functions like sigmoid.

Finally, it is important to note that overfitting would lead to unreliable predictions, and it is, therefore, unlikely to have affected our results, given the prediction success after a few iterations. Therefore, we conclude that the safeguards listed above efficiently limited overfitting.

### 2.5. The Impact of Missing Data, False Positives, and False Negatives on the AI-Iterative Approaches

In this work, we analyzed high-quality datasets from previous studies [9,24]. The sets essentially span the full combinatorial space of the targeted mutational changes and very few data are missing (i.e., very few variants lack experimental fitness or antibody evasion data). Furthermore, the control experiments reported in the original publications [9,24] disfavor a substantial proportion of outliers, false positives, and false negatives. Consequently, we ignored missing data with no relevant consequences for our analyses. Moreover, there is no clear-cut rule to determine a priori which data points must be considered as outliers, false positives, and false negatives. Overall, in order to avoid biases, we decided to draw the training sets from the complete sets of experimental data.

Certainly, poor-quality data, including a large proportion of erroneous data, would have compromised our analyses, and any kind of analysis for that matter (“garbage in, garbage out”). However, the iterative nature of our protocol should have immediately revealed such a scenario, as faulty experimental protocols for variant characterization would lead to increasingly erroneous prediction in successive iterations.

## 3. Results

### 3.1. Searching the Sequence Space of the Enzyme Dihydrofolate Reductase

Papkou et al. [19] recently used CRISP-Cas9 deep mutagenesis to randomize 9 positions in the gene of the enzyme dihydrofolate reductase. The randomized positions correspond to the codons for three consecutive amino acid residues located at the enzyme's active site (see Figure 2a). Randomizing the 9 positions resulted in a library of ~260,000 nucleotide sequences and DNA genotypes. Papkou et al. [19] assigned experimental fitness values to essentially all of the DNA sequences in the library. In evolutionary biology, the concept “fitness” is commonly used describe the organism’s capability to survive or reproduce. In studies on enzyme engineering and molecular evolution, on the other hand, it typically refers to a protein’s biomolecular property (e.g., activity or stability) that it is expected to be related to organism survival [1]. However, in the case of the data reported by Papkou et al. [19], the fitness values were actually defined in terms of organismal survival, as they were derived from DNA variant frequencies in the organism’s population. Specifically, these authors expressed the library in *E. coli*, exposed the resulting *E. coli* population to the antibiotic trimethoprim, potentially degraded by active variants of the enzyme, and performed deep scanning mutagenesis to determine variant frequencies after selection. Numerical fitness values were then calculated as the variant frequencies in a natural logarithm scale, with the wild-type enzyme taken as the reference and assigned a fitness value of zero. Each fitness value thus calculated is a metric of the contribution of the variant to the survival or reproduction of the hosting cells and may reflect a more or less complex combination of the relevant biomolecular properties. First, since the library spans 3 positions in the amino acid sequence and three codons in the 9 positions of the gene sequence, there are 8000 possible amino acid sequences that may encode for proteins that differ in features that obviously affect fitness, such as the activity and folding efficiency. However, fitness effects also reflect variant expression levels, which may directly depend on the nucleotide sequence [19], and two identical amino sequences encoded by different DNA sequences may, thus, display different fitness values. Consequently, our analyses involved a search in a fitness landscape of ~260,000 individual DNA sequences. Note that regardless of complex molecular interpretations of fitness in this case, the availability of enzyme fitness values for a library of ~260,000 variants allowed us to test the approach proposed in this work in the context of enzyme optimization.

Figure 2b shows the distribution of fitness values for the ~260,000 variants of the combinatorial library. The distribution peaks at negative values of about −0.7, indicating that most variants in the library are substantially worse than the wild type (which was assigned a fitness value of zero). This result likely reflects that for many variants, proper folding is compromised, enzyme activity is impaired, or expression levels are greatly diminished. A small fraction of the variants displays enhanced fitness with respect to the wild type, and only about 0.7% of the variants have fitness values of unity or above, as shown in the lower panel of Figure 2b. We selected fitness > 1 as the optimization target for the analyses described below.

The sequence and fitness value of the wild-type enzyme is, of course, known. However, our analyses were meant to represent the common experimental scenario in which no information about the properties of the library variants is a priori available. Therefore, we ignored any previous knowledge and started searches by randomly selecting 20 variants. The chosen AI tool (see Figure 1a,c,d) was then trained on the sequence/fitness data for these 20 variants and the trained tool was then used to predict fitness values for all variants in the combinatorial space (4^9^ = 262,144 sequences). The sequence/fitness data for the top 20 predictions for which experimental fitness values are available were then incorporated into the training set for the next round, thereby initiating the iterative learning procedure. An illustrative example of this protocol is shown in Figure 2c. Initially, the fitness values are low, reflecting the distribution of the fitness values for the whole library (Figure 2b). However, after only 5 rounds of iterative training involving a total of only 120 variants, the 20 top predictions already display examples of sequences that meet our optimization target, i.e., sequences with experimental fitness above unity.

To explore the extent to which the success of the iterative search process depends on the starting set of 20 variants, we performed, for each of the three types of AI tools used, 1000 replicas of the protocol described above. We focused the analysis of the results obtained on the best variant from each search, in congruence with the common protein biotechnology scenario in which a practical application may be enabled by a single variant with the required enhanced properties. Figure 3a shows the distribution of the best variants at the fifth round. A substantial fraction of the searches meets the proposed target (fitness higher than unity) at the fifth iteration (i.e., after training with a total of 120 variants), with the performance of the different types of AI tools following the ranking GXBoost > random forest > multi-layer perceptron (Figure 3a). The success statistics are substantially improved if instead of a single AI tool we consider ensembles of 5 AI tools of the same type, each one being independently trained. As shown in Figure 3b, most of the ensembles meet the optimization target (fitness higher than unity) at the fifth iteration, which involves in this case training with a total of 5 × 120 = 600 variants. Actually, in the fifth iteration, most of the ensembles achieve fitness values higher than 1.2, which corresponds to a tiny fraction of the total number of sequences considered (Figure 2b).

It must be noted at this point that each trained AI tool does not (and it is not meant to) predict experimental fitness for the whole combinatorial library to any significant degree of accuracy. Rather, each AI tool approaches a given peak in the fitness landscape, with different successful tools approaching different peaks; that is, different searches lead to different sets of high-fitness variants, as it is illustrated in Figure 3c.

Finally, it is of interest to assess the improvement afforded by the AI iterative approach with respect to a non-focused, random library screening. It must be noted in this context that for many enzyme engineering applications, a single variant with the desired enhanced properties may suffice to enable a biotechnological application. We, therefore, based our assessment on computational estimates of the number of variants that need to be screened (i.e., the required screening effort) to obtain at least one variant that meets the optimization target. For the illustrative purposes of our analyses, we selected fitness ≥ 1.2 (see Figure 2b) as the target. There were only 290 variants with fitness values equal to or higher than 1.2; that is, only about 0.1% of the total size of the combinatorial library. To simulate non-focused screening, we randomly drew variants from the library until a variant meeting the target was extracted. We performed 1000 replicas of this stochastic process in such a way that we could derive a profile of the probability of drawing at least one variant meeting the target versus the number of variants screened. The profile is shown in Figure 3d, where it is apparent that more than 1000 need to be screened to reach large probabilities of obtaining the target fitness level. In contrast, a simple calculation shows that the iterative AI approach leads to high probabilities upon a total screening effort of approximately 100 variants (see Figure 3d), implying a decrease of approximately one order of magnitude in screening effort. This would represent a substantial improvement, in particular if methodologies to exhaustively screen large libraries are not available, which is likely to be the case for many biotechnologically relevant goals, such as the enhancement of a low-level promiscuous activity or the engineering of enzyme regio- and stereo-selectivity.

### 3.2. Searching the Sequence Space of the Receptor-Binding Domain of SARS-CoV-2

**Figure 4:**
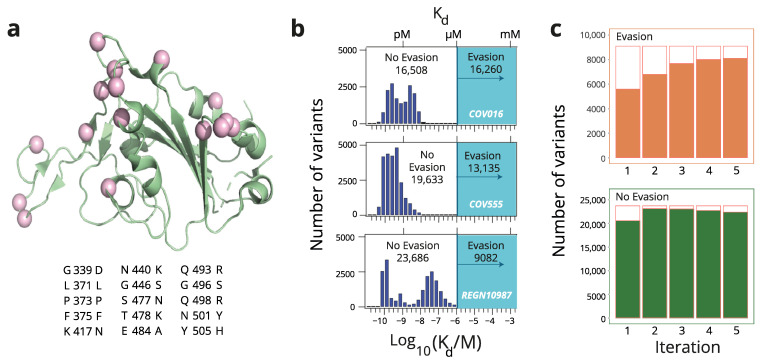
Distribution of antibody affinity to variants of the receptor-binding domain (RBD) of the SARS-CoV-2 virus. Recently [24], yeast display and flow cytometry were used to determine binding affinities of several monoclonal antibodies to the variants in a complete combinatorial library including all RBD intermediates between the parent Wuhan Hu-1 strain and the Omicron strain. (**a**) The 3D structure of the RBD (PDB ID 7BH9) showing the positions at which there are amino acid differences between the RBD of the Omicron BA.1 strain and that of the parent Wuhan Hu-1 strain. The specific amino acid replacements are listed below the structure. The combination of these replacements gives rise to a library of 2^15^ = 32,768 variants. (**b**) Distribution of antibody–RBD dissociation constants for the variants of the library and three different neutralizing antibodies: LY-CoV016, LY-CoV555, REGN10987 [24]. Dissociation constants higher than 1 micromolar could not be determined experimentally and are taken to define antibody evasion. Numbers of evading and non-evading variants for each antibody are shown. (**c**) Illustrative example of an AI-driven iterative learning search of the library. XGBoost (Figure 1d) was initially trained with 20 randomly chosen variants and predictions were used to increase the training set, with 20 additional variants at each iteration round. The plots show the total number evading and non-evading variants for the antibody REGN10987, as well the number of correct predictions in each case (solid color).

The receptor-binding domain (RBD) of the spike protein provides the major antigenic target of the SARS-CoV-2 virus. Fifteen mutations separate the RBD of the Omicron BA.1 strain from that of the parent Wuhan Hu-1 (see Figure 4a). Moulana et al. [24] generated a complete combinatorial library including all RBD intermediates between the parent and the Omicron strain, i.e., 2^15^ = 32,768 amino acid sequences. They displayed the library on the surfaces of yeast and used flow cytometry coupled to sequencing to determine the binding affinities of several monoclonal antibodies to most of the RBD variants in the library. These included three neutralizing antibodies (LY-CoV016, LY-CoV555, REGN10987) that have been used in the treatment of COVID-19, although the three of them are evaded by the Omicron strain. The availability of antibody dissociation constants for a library of about ~30,000 RBD variants allowed us to test our approach in the context of antibody evasion.

Distributions for the logarithms of the experimental antibody RBD dissociation constants are shown in Figure 3b. Many dissociation constant values are much smaller than 1 micromolar, indicating tight antibody–RBD binding. However, for a substantial number of variants, the dissociation constants are larger than 1 micromolar and could not be determined with the methodology used here [24]. These variants do not bind the antibodies, or at least they bind them weakly and likely non-specifically [24]. We took the range of dissociation constants of 1 μM and above to correspond to antibody evasion (Figure 3b). Under this definition of evasion, 50%, 40%, and 28% of the RBD variants evade the antibodies LY-CoV016, LY-CoV555, and REGN10987, respectively (Figure 3b).

We aimed to show that an iterative learning approach capable of efficiently classifying the sequences in two classes, evading and not evading, for each given antibody. The process was started by randomly selecting 20 variants. The selected AI tool was then trained on the sequence/evasion data (https://github.com/desai-lab/omicron_ab_landscape, accessed 2 November 2024) for these 20 variants and the trained tool was used to classify all variants in the combinatorial space (2^15^ = 32,768) into the two specified classes. To avoid losing variant diversity in the training set, the 20 variants incorporated into each subsequent round of training were selected to have a minimum number of sequence differences (typically 3, see Methods for details) with those in the previous training set. As an illustration of the approach, Figure 4c shows the result of the training of the XGBoost tool to classify the REGN10987 evasion data (https://github.com/desai-lab/omicron_ab_landscape, accessed 2 November 2024). After only five rounds of iterative training (involving a total of 120 variants in the sequence set), most of the ~30,000 sequences were correctly classified into the evasion versus non-evasion classes.

**Figure 5:**
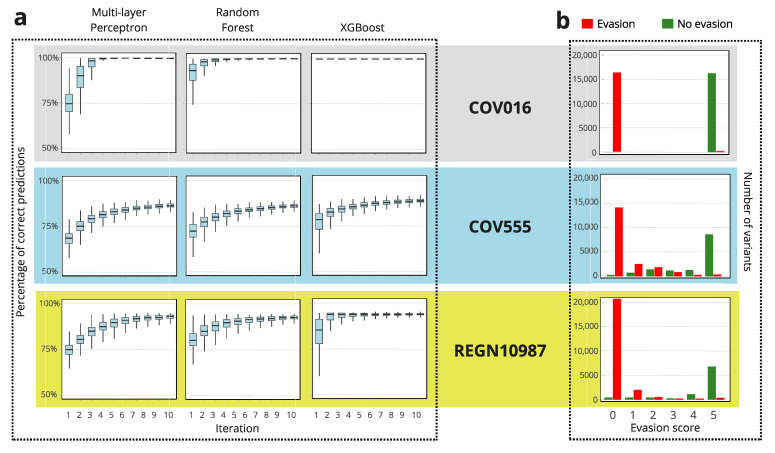
Using AI-driven iterative learning to classify RBD variants into evading and non-evading classes. The AI tools displayed in Figure 1 were iteratively trained on small sets of variants randomly selected from the original set of experimental data on antibody evasion [24]. In order to test the robustness of the approach, a large number of replica analyses were performed. (**a**) For each antibody or AI type, 1000 replica searches were performed. The results are displayed as box-and-whisker plots of the percentage of correct predictions (i.e., correct classification of the variant as evading or non-evading for the considered antibody). The average success in variant classification in the 5th iteration is generally higher than 80%, even reaching ~100% in some cases. (**b**) Illustration of a simple approach to assess the reliability of the prediction (i.e., the classification as evading or non-evading) for each given sequence. An ensemble of five XGBoost models was used to search the RBD sequence space for evasion or non-evasion of the three antibodies studied: LY-CoV016, LY-CoV555, REGN10987. An evasion score between 0 and 5 was assigned to each sequence as the number of times the variant was predicted to evade the antibody at the fifth iteration. For the three antibodies, most variants were assigned scores of 5 and 0. With very few exceptions, variants with a score of 5 are found experimentally to evade the antibody, while variants with a score of 0 do not evade the antibody, again with very few exceptions.

To explore the extent to which the success of the iterative search process depends on the starting set of 20 variants, we performed 500 replicas of the protocol described above for each of the three types of AI tools used (Figure 1) and with the three antibodies studied (Figure 4b). The results are displayed as box-and-whisker plots in Figure 5a. Even after 5 iterations (a total of 120 variants in the training set), the average success rate in variant classification (evasion versus no evasion) was higher than 80% and actually approached 100% in some cases. The performance of the different types of AI tools appeared again to follow the ranking XGBoost > random forest > multi-layer perceptron. Furthermore, it emerged that the efficiency of the search also depends on the antibody considered, and the ranking LY-CoV016 > REGN10987 > LY-CoV555 is visually apparent in Figure 5a.

Predictions can never be perfect and must be taken with due caution, in particular when dealing with predictions on antibody evasion. Therefore, it is of interest in this context to propose methods to derive a metric of the reliability of the prediction (i.e., the classification as evading or non-evading) for each given sequence. Figure 5b illustrates a simple, practical approach to achieve this. An ensemble of five XGBoost models was used to search the RBD sequence space for evasion or non-evasion of the three antibodies studied: LY-CoV016, LY-CoV555, REGN10987. An evasion score of between 0 and 5 was assigned to each sequence as the number of times the sequence was predicted to evade the antibody at the fifth iteration. The approach, therefore, involved a total of 5 × 120 = 600 sequences in the training set. With very few exceptions (see Figure 5b), all sequences with a score of 5 actually evade the antibody and all sequences with a score of 0 do not evade the antibody. The analysis may also provide an assessment of the overall reliability of the classification for each given antibody, in terms of the number of sequences with intermediate scores (1–4). This number again follows the ranking LY-CoV016 > REGN10987 > LY-CoV555 (Figure 5b).

## 4. Discussion

We have provided evidence that libraries of at least a few hundred thousand variants can be efficiently searched for much enhanced protein properties using state-of-the-art AI tools that are iteratively trained on a much smaller number of variants. To this end, we analyzed fitness data on a library of ~260,000 variants of the enzyme dihydrofolate reductase, which have been recently determined using a specialized methodology based on in vivo selection [19]. Our approach identifies the scarce, high-fitness variants of the enzyme from iterative training based on just a few hundred variants. This is a remarkable result because of the prevalence of epistasis in the data [19], including higher-order epistasis (i.e., non-additivity of the effects of more than two mutations), which results in a rugged and challenging fitness landscape to search.

To test the generality of our proposed approach, we applied it to a completely different problem—the emergence of the receptor-binding domain (RBD) of the Omicron BA.1 strain of the SARS-CoV-2 virus from that of the parent Wuhan Hu-1 strain. We took advantage of the fact that recently [24], specialized methodologies have been used to determine antibody affinities to essentially all RBD intermediates between the parent and the Omicron strain, i.e., a library of ~30,000 variants. Despite the fact that pervasive epistasis determines antibody affinity in this case [24], we found that upon iterative training on a total of just a few hundred variants, the AI tools correctly assigned most variants to the evasion or non-evasion classes. This result supports the potential of our approach for the experimental assessment of how viral proteins may evolve in response to antiviral strategies.

The approach we have demonstrated in this work is efficient, first in terms of the computational effort involved, as each search takes only a few seconds on a CPU-based personal computer. It is important to note, furthermore, that we used the default parameters of the AI tools employed (see Methods for details); that is, we did not optimize the AI tools for the specific problems we addressed, which further supports the generality of the approach proposed here.

Most importantly, the approach we developed in this work is also efficient in terms of the number of variants required for iterative training (“learning”). The training sets in the simulations reported here were, thus, increased by 20 variants at each iteration, and in most cases the optimization target was already met in the 5th iteration. Additionally, some analyses were based on ensembles of five independently trained AI tools, involving a total 100 variants per iteration. We actually selected these numbers (20 and 20 × 5) on purpose, because they correspond to numbers of variants that can be experimentally studied in the lab, even if specialized methodologies for high-throughput screening are not available. For instance, sets of 100 gene fragments of specified sequences can be ordered from various companies, then subsequently cloned and screened as a small library for the desired enzyme property.

Overall, this paper presents an efficient, widely applicable, low-throughput experimental approach to enzyme optimization and the prediction of antibody evasion patterns. However, it is important in this context to discuss the factors that could limit the general applicability of the approach. Overfitting during training could in principle compromise the performance of the AI-driven iterative approach. However, besides the fact that we used regularization and other procedures to limit this possibility (see Materials and Methods for details), overfitting would be easily detected as it would lead to unreliable predictions (for instance, predicted fitness values much higher than the experimental ones). Overfitting is, therefore, unlikely to have affected our results, given the prediction success after a few iterations. Another factor that could potentially compromise the approach is a strong dependence of the results on the initial sets of variants. However, our extensive replica analyses provide a reliable test of this possibility; in fact, the consistency and robustness of the results obtained here argues against a strong dependence on the initial sets. On the other hand, it must be recognized that the scarcity of complete or almost complete experimental data on large variant libraries precludes an exhaustive test of the approach at this stage. However, while a full assessment of the approach must wait for future experimental work, its success with the two quite different protein systems and two quite different problems, as reported in this work and as demonstrated in our manuscript, makes us optimistic in this regard.

## Data Availability

Analyses were performed with custom code deposited with Github: https://github.com/isasio/Virus-Enzyme-Prediction (accessed on 2 November 2024).
